# Associations between increased intervention coverage for mothers and newborns and the number and quality of contacts between families and health workers: An analysis of cluster level repeat cross sectional survey data in Ethiopia

**DOI:** 10.1371/journal.pone.0199937

**Published:** 2018-08-02

**Authors:** Elizabeth Allen, Joanna Schellenberg, Della Berhanu, Simon Cousens, Tanya Marchant

**Affiliations:** London School of Hygiene and Tropical Medicine, London, United Kingdom; BRAC, BANGLADESH

## Abstract

**Background:**

Survival of mothers and newborns depends on life-saving interventions reaching those in need. Recent evidence suggests that indicators of contact with health services are poor proxies for measures of coverage of life saving care and attention has shifted towards the quality of care provided during contacts.

**Methods and findings:**

Regression analysis using data from representative cluster-based household surveys and surveys of the frontline health workers and primary health facilities in four regions of Ethiopia in 2012 and 2015 was used to explore associations between increased numbers of contacts or improvements in quality and any change in the coverage of interventions (intervention coverage). In pregnancy, in multiple regression, an increase in the quality indicator ‘focused ANC behaviours’ was associated with a change in both the coverage of iron supplementation and syphilis prevention ((regression coefficients (95% CI)) 0·06 (0·01, 0·11); 0·07 (0·04, 0·10)). This equates to a 0.6% increase in the proportion of women taking iron supplementation and a 0.7% in women receiving syphilis prevention for a 10% increase in the quality indicator ‘focused ANC behaviours’. At delivery, in multiple regression the quality indicator ‘availability of uterotonic supplies amongst birth attendants’ was associated with improved coverage of prophylactic uterotonics (0·72 (0·50, 0·94)). No evidence of any relationships between contacts, quality and intervention coverage were observed within the early postnatal period.

**Conclusions:**

Increases in both contacts and in quality of care are needed to increase the coverage of life saving interventions. For interventions that need to be delivered at multiple visits, such as antenatal vaccination, increasing the number of contacts had the strongest association with coverage. For those relying on a single point of contact, such as those delivered at birth, we found strong evidence to support current commitments to invest in both input and process quality.

## Introduction

Improving the survival of mothers and newborns depends on life-saving interventions reaching those in need [1]. In the context of maternal and newborn health in high-mortality settings these interventions include both biomedical interventions delivered by health care workers such as the use of prophylactic uterotonics during the third stage of labour and behaviours practised by health care workers or by families such as avoiding newborn infection by putting nothing harmful on the newborn cord.

Many of the interventions best delivered in a health facility by skilled health workers (doctor, nurse, midwife) are difficult to measure during household surveys because families interviewed cannot be assumed to provide reliable reports, especially for events around the time of birth [2, 3]. Consequently, maternal and newborn measurement practice has been to use indicators of contact with health services at different points along the continuum from pregnancy to postnatal period as proxy measures for the coverage of life saving care. However, a growing body of evidence suggests that such contact coverage estimates are poor proxies for life saving effects [4]. Attention has therefore shifted towards the quality of care and interventions delivered during contacts [5, 6].

Ethiopia is a country with ambitious targets to reduce maternal and newborn mortality. The Ethiopian Federal Ministry of Health (FMOH) 2015 “Health Sector Transformation Plan” [7] committed to reduce the country’s maternal mortality ratio from 420/100,000 live births in 2013 to 199/100,000 in 2020, and the neonatal mortality rate from 28/1,000 live births in 2013 to 10/1,000 in 2020. It describes the pathway to achieving these targets as including near universal coverage of at least four antenatal care visits, skilled attendance at delivery, and postnatal care for every pregnant woman and her newborn. This plan builds on the 2003 “Accelerated Expansion of Primary Health Care Coverage,” comprehensive Health Extension Program (HEP) which recognised the huge gap between need and availability of health care services in the country. Now, in recognition of the importance of quality delivery care [8], the 2015 Health Sector Transformation Plan specifically targets improvement in the quality of care provided to mothers and newborns in addition to enhancing demand and increasing availability.

In this context of rapid improvement from low coverage of contacts between families and the health system [9] at the same time as strengthening the availability of quality life-saving care in facilities [10], evidence from Ethiopia can provide important insights into the relationship between changes in contacts, quality and intervention coverage for mothers and newborns. Using linked household survey data and skilled birth attendant interviews from four regions of Ethiopia collected in 2012 and in 2015 (DOI: 10.17037/DATA.129), this study aimed to strengthen the evidence base on the importance of quality of contacts between families and health workers in addition to the role of the number of contacts alone. We examined the associations between increases in contact coverage and increases in quality of care and how these are associated with the coverage of life saving interventions in Ethiopia during this time. Details of contact and quality indicators and lifesaving interventions are given in the methods.

## Methods

This work was a secondary analysis carried out as part of a programme of research to understand what works, where and how to improve maternal and newborn health in selected high-mortality settings [11]. In Ethiopia, representative cluster-based household surveys and surveys of the frontline health workers and primary health facilities assigned to provide routine maternal and newborn health services to those households were performed in the four regions of Oromia, Tigray, Amhara and Southern Nations Nationalities and Peoples (SNNP) in 2012 and again in 2015. In Ethiopia, primary care is organized at the *woreda* (district) level within primary healthcare units which each include a health centre and several rural health posts where Health Extension Workers provide basic services and refer patients in need, including for care at birth. In addition, a primary hospital provides referral care within each *woreda*. Health Extension Workers connect with communities through local volunteers known as the Women’s Development Army.

### Data collection methods

The survey included household and facility surveys linked at the cluster level. Data collection methods used in 2012 have previously been described [2]. The same methods were applied in 2015, including returning to the same clusters. Each survey was treated as a cross sectional with no attempt made to repeat or to avoid interviews with the same individual women. Ethiopia is organised by region, zone, *woreda* (district), *kebele* (similar to a ward; the lowest level of census population data) and *gote* (proxy for village). The 2012 survey included 80 clusters which were sampled from 76 *woreda* across the four regions. Sampling of clusters was performed by listing all *woreda* geographically from north to south of the country, listing *kebeles* and their population size alphabetically within each *woreda*, and systematically sampling 80 *kebeles* with probability proportional to population size. *Gotes* within each of these 80 *kebele* were listed and one *gote* per *kebele* selected using simple random sampling. For the household survey, at each selected *gote*, all households were listed and *gotes* segmented into groups of 75 or fewer households: field teams randomly selected one segment from each *gote* as the cluster to be surveyed. All households within each selected cluster were visited and all resident women aged 13–49 who had given birth in the last 12 months interviewed using a modular questionnaire that included information about the demographics of the household, recent birth history, and experience of care around the time of the most recent birth.

For the facility survey, at each sampled cluster, the health centre allocated to provide routine antenatal, intrapartum and postnatal care to the selected cluster was surveyed for facility readiness, including information about stocks and supplies, staffing, and the volume of events taking place in that facility. In addition, the staff member who attended the last delivery recorded in the maternity register was interviewed about that birth event. All birth attendants were eligible for this interview which was designed to reflect the most recent birth experience in the facility. The sample size for the surveys in each year are shown in Table 1. The same health centres were surveyed at both time points.

**Table 1:** Size of the surveys accessed in this analysis.

	May 2012	May 2015
Number of household clusters sampled across four Regions^1^	80	80
Total number of households surveyed^2^	4294	6000
Total number of resident women aged 13–49 interviewed	3937	6510
Number with a birth in the 12 months prior to survey^3^	533	787
Number of birth attendant interviews in primary health facilities^4^	316	310

^1^ The 2015 survey teams returned to precisely the same geographical location as surveyed in 2012

^2^ The cluster size was increased from 50 households in 2012 to 75 households per cluster in 2015

^3^ In 2012 no women with a birth in the 12 months prior to survey were identified in 1 household cluster (included in analysis n = 79)

^4^ Used for linking with household interviews, linked by cadre of health worker each woman reported having contact with

This led to two sources of data for this analysis; household data and birth attendant data collected during the facility survey. All coverage estimates for contacts and lifesaving interventions are population level estimates, derived from the household survey. Measures of input and process quality were derived from the birth attendant data and incorporated by linking individual women’s reports from the household surveys to data about the cadre of the health worker who provided care to the woman.

### Hypotheses and covariates

The research question that led to the secondary analysis of the data was whether a change in the coverage of life saving interventions had occurred over time, and what the relative contribution of an increased number of contacts or improvements in quality had been to such change. An analysis plan was written in 2015 prior to analysing the data (**S1 File Prospective Analysis Plan**), informed by consensus in the global literature about the need to increase coverage of life saving interventions delivered to mothers and newborns by frontline health workers [11, 12]. For antenatal care, intrapartum care, and postnatal care we defined key indicators of contact, quality, and intervention coverage (Table 2) that could plausibly be hypothesised to lie along one pathway (Table 3). For example, in pregnancy we hypothesised that an increase in coverage of iron supplementation could be achieved through an increase in number of ANC contacts, or through an increase in number of health worker behaviours for focussed ANC (Table 2), independently of the number of visits made, or some combination of both. For the intrapartum period, we hypothesised that an increase in coverage of prophylactic uterotonics during the third stage of labour could be achieved through an increase in coverage of skilled birth attendance; doctor, nurse, midwife, or through an increase in availability of uterotonics amongst all birth attendants, or some combination of both. During the postnatal period, we hypothesised that an increase in exclusive breastfeeding for the first 3 days of life could be achieved through an increase in coverage of postnatal care within 2 days of birth, or through an increase in knowledge of the importance of breastfeeding amongst frontline workers making postnatal care visits, or some combination of both. Across stages in the continuum from pregnancy to the postnatal period, we hypothesised that an increase in clean cord care could be achieved through an increase in coverage of ANC, or through an increase in appropriate birth preparations made by the mother, or some combination of both. Details of the creation of composite indicators are given in Table 2.

**Table 2:** Cluster level summaries of the indicators for contacts, quality and lifesaving interventions across the continuum of care in 2012 and 2015 amongst women with a live birth in the 12 months preceding survey.

		Indicator	Definition	Data source	Components	2012	2015
						Point estimate (95% CI)	Point estimate (95% CI)
PREGNANCY	Contacts	Coverage of at least 4 ANC visits	Proportion of women who had at least 4 pregnancy care interactions	Household Survey		22 (17, 27)	45 (40, 50)
	Quality	Focused ANC (components delivered)	Mean number of health worker behaviours for good quality antenatal care received by end of pregnancy	Household Survey	8 health worker behaviours of focused antenatal care including weight, height, blood pressure measured, urine and blood tested, counselled on danger signs, birth preparedness and breastfeeding	2·8 (2·4, 3·2)	3·7 (3·4, 4·0)
		Birth preparedness (number of items prepared)	Mean number of appropriate preparations for their delivery made while still pregnant	Household Survey	5 preparations including finances, transport, food and identified a birth attendant and a facility	2·5 (2·3, 2·8)	2 (1·8, 2·2)
	Interventions	Coverage of iron supplementation	Proportion of women who received iron supplementation during pregnancy	Household Survey	Any iron supplementation received during pregnancy	16 (13, 20)	41 (36, 46)
		Coverage of tetanus toxoid protection	Proportion of women who received tetanus toxoid vaccination: effective protection	Household Survey	Two doses last three years or five in lifetime	40 (34, 46)	41 (35, 46)
		Coverage of syphilis detection	Proportion of women who received syphilis test results from ANC blood test	Household Survey		8 (4, 11)	13 (10, 17)
INTRAPARTUM	Contacts	Coverage of skilled attendant at birth	Proportion of women who were attended by a skilled attendant during delivery	Household Survey		18 (12, 23)	51 (44, 58)
	Quality	Knowledge of thermal care amongst birth attendants	Proportion of women who were attended at birth by an attendant who had appropriate knowledge of thermal care	Linked household and birth attendant survey	In unprompted knowledge assessment birth attendant reported importance of making sure the baby is kept warm at birth (skin to skin/kangaroo technique/thermal care)	58 (47, 70)	64 (54, 74)
		Knowledge of management of heavy bleeding amongst birth attendants	Mean number of components of how to treat heavy bleeding of FLWs attending birth known	Birth attendant survey	Mean score from unprompted knowledge assessment including: give uterotonics; begin iv fluids; empty full bladder; take blood for haemoglobin and cross matching; examine woman for lacerations; manually remove retained products; refer	2·1 (1·8, 2·5)	3·6 (3·2, 4.0)
		Knowledge of importance of breastfeeding amongst birth attendants	Proportion of women attended at birth by an attendant who had appropriate knowledge of breastfeeding	Linked household and birth attendant survey	In unprompted knowledge assessment birth attendant reported importance of at least one of: promote breastfeeding; provide extra support to the mother to establish breastfeeding; monitor ability to breastfeed	93 (88, 98)	76 (67, 85)
		Availability of uterotonics at birth	Proportion of women attended by any birth attendant who had uterotonics available	Linked household and birth attendant survey	One of either oxytocin, ergometrine, misoprostol, or syntometrine available	32 (22, 42)	69 (61, 77)
	Interventions	Coverage of prophylactic uterotonics to prevent post-partum haemorrhage	Proportion of women who received a prophylactic uterotonic immediately after birth	Linked household and birth attendant survey		21 (12, 31)	64 (55, 73)
		Coverage of immediate drying	Proportion of babies immediately dried	Household survey	Dried within five minutes	20 (15, 25)	22 (17, 26)
		Coverage of immediate wrapping	Proportion of babies immediately wrapped	Household survey	Wrapped within five minutes	27 (20, 33)	38 (32, 43)
		Coverage of delayed bathing	Proportion of babies with delayed bathing	Household survey	Bathed after 24 hours	37 (31, 44)	54 (48, 60)
		Coverage of immediate breastfeeding	Proportion of babies immediately breastfed	Household survey	Within one hour	44 (38, 51)	62 (57, 67)
		Coverage of hand washing by birth attendants	Proportion of home deliveries where birth attendant washed hands	Household survey	With soap (home births)	78 (72, 84)	61 (52, 71)
		Coverage of clean cord care	Proportion of newborns who had clean cord care	Household survey	Sterile cutting, tying and nothing harmful (chlorhexidine permitted) put on the cord after birth	42 (34, 49)	36 (30, 43)
POSTNATAL	Contacts	Coverage of postnatal visit within 2 days	Proportion of women who reported their newborn had a PNC (any location) within 2 days of birth	Household survey		3 (1, 5)	6 (4, 8)
	Quality	Recommended PNC components carried out	Mean number of components of good quality PNC received	Household survey	Definition of good quality post-natal care includes: baby weighed, checked for clean cord care, danger signs, caregiver counselled on bf and thermal care	0·2 (0·1, 0·2)	0·2 (0·1, 0·3)
	Interventions	Exclusive breastfeeding during first three days of life	Proportion of women practising exclusive breastfeeding for three days	Household survey		85 (80,91)	92 (89, 95)
		Nothing harmful put on newborn cord	Proportion of women putting nothing harmful on the cord	Household survey		78 (72, 83)	90 (87, 93)

**Table 3:** Hypothesised pathways and simple regression analysis.

	Hypothesised pathway	Indicators of contacts and quality on coverage of stated intervention:	Coverage of stated intervention:	Coefficient (95% CI)	p value
PREGNANCY	**Contact:** More antenatal interactions **Quality:** 1. more components of focused ANC being provided 2. better birth preparedness carried out **Coverage:** higher coverage of biomedical interventions received	**Contact:** least 4 ANC interactions	*Iron supplementation*	0·33 (0·10, 0·56)	0·006
			*tetanus toxoid protection*	0·37 (0·14, 0·60)	0·002
			*syphilis detection*	0·28 (0·13, 0·43)	<0·001
		**Quality:** Focused ANC (health worker behaviours delivered)	*iron supplementation*	0·07 (0·04, 0·11)	<0·001
			*tetanus toxoid protection*	0·06 (0·02, 0·09)	0·005
			*syphilis detection*	0·07 (0·05, 0·09)	<0·001
		**Quality:** Birth preparedness (number of items prepared)	*iron supplementation*	0·19 (0·09, 0·29)	<0·001
			*tetanus toxoid protection*	0·12 (0·02, 0·23)	0·024
			*syphilis detection*	0·14 (0·08, 0·21)	<0·001
INTRAPARTUM	**Contact:** More deliveries with a skilled birth attendant **Quality**: better exposure to knowledge about the importance of breastfeeding **Coverage**: higher coverage of immediate breastfeeding	**Contact**: skilled attendant at birth	*immediate breastfeeding*	0·03 (-0·15, 0·21)	0·722
		**Quality**: knowledge about the importance of breastfeeding	*immediate breastfeeding*	0·05 (-0·09, 0·18)	0·504
	**Contact:** More deliveries with a skilled birth attendant **Quality**: better exposure to knowledge about the importance of newborn thermal care **Coverage**: higher coverage of immediate thermal care	**Contact**: skilled attendant at birth	*immediate drying*	0·10 (-0·06, 0·27)	0·220
			*immediate wrapping*	0·17 (-0·02, 0·36)	0·084
			*delayed bathing*	0·41 (0·22, 0·59)	<0·001
		**Quality**: knowledge about the importance of newborn thermal care	*immediate drying*	0·02 (-0·10, 0·14)	0·750
			*immediate wrapping*	0·001 (-0·13, 0·14)	0·984
			*delayed bathing*	0·05 (-0·10, 0·21)	0·478
	**Contact**: More deliveries with a skilled birth attendant **Quality**:1. better knowledge about management of heavy bleeding2. better exposure to uterotonic supplies **Coverage**: higher coverage of prophylactic uterotonics	**Contact**: skilled attendant at birth	*prophylactic uterotonics*	0·61 (0·29, 0·93)	<0·001
		**Quality**: knowledge about management of heavy bleeding	*prophylactic uterotonics*	0·09 (0·04, 0·14)	0·001
		**Quality**: supplies of uterotonics for birth attendants	*prophylactic uterotonics*	0·84 (0·66, 1·02)	<0·001
POSTNATAL	**Contact**: More postnatal contact **Quality**: more components of recommended PNC being provided **Coverage**: higher coverage of exclusive breastfeeding for the first 3 days of life and higher coverage of nothing harmful applied to the newborn cord	**Contact**: postnatal visit within 2 days	*exclusive breastfeeding*	0·03 (-0·32, 0·38)	0·870
			*nothing harmful on cord*	0·13 (-0·21, 0·46)	0·446
		**Quality**: Recommended PNC (components carried out)	*exclusive breastfeeding*	0·05 (-0·06, 0·17)	0·363
			*nothing harmful on cord*	0·10 (-0·01, 0·20)	0·072
ACROSS STAGES	**Contact**: more antenatal contacts **Quality**: better birth preparedness carried out **Coverage**: higher coverage of intrapartum (hand washing with soap by birth attendant for home birth) and newborn (clean cord care) interventions	**Contact:** least 4 ANC interactions	*hand washing by birth attendant*	0·14 (-0·33, 0·61)	0·546
			*clean cord care during birth*	-0·28 (-0·56, 0·001)	0·053
		**Quality**: Birth preparedness (number of items prepared)	*hand washing by birth attendant*	0·19 (-0·05, 0·43)	0·120
			*clean cord care during birth*	-0·10 (-0·23, 0·03)	0·130

### Statistical analysis

Cluster level summaries (proportions for binary indicators and means for continuous indicators) of the indicators for ‘contact’, ‘quality’ and ‘intervention coverage’ were calculated in 2012 (baseline) and 2015 (endline) (Table 2).

Initial simple regression analyses were carried out using linear regression, regressing the cluster level mean difference in ‘intervention coverage’ indicators on the cluster level mean difference in indicators of ‘contact’ and ‘quality’ between 2012–2015. The analysis adjusted for baseline cluster level summaries of ‘contacts’ or ‘quality’ and of ‘coverage of critical interventions’ (equation 1). Analysis was restricted to the hypothesised relationships between change in contacts, quality and interventions described in Table 3. Multiple linear regression models were then fitted regressing the cluster level mean difference in ‘intervention coverage’ indicators on the mean difference in any indicators of ‘contact’ and ‘quality’ that had shown an association in the initial analysis. Again, all analysis adjusted for baseline cluster level summaries of both ‘contacts’ or ‘quality’ and of ‘intervention coverage’.

Analysis was at the cluster level and included all 80 clusters present at baseline and endline. All analysis was done in Stata 14 [13].

Equations used were of the form ‘cluster level change in intervention coverage between 2012 and 2105’ = ‘cluster change in quality or contact indicator between 2012 and 2105’ + ‘2012 (baseline) intervention coverage’ + ‘2012 (baseline) quality or contact indicator’.

### Ethics

In Ethiopia, national level support was obtained from the Ministry of Health in Ethiopia, and ethical approval from the Ministry of Science and Technology; at the Regional level, approval was granted by the Regional IRBs in Amhara, Oromia, SNNP, and Tigray. Written informed consent was obtained from all participants and the information provided included description of this analysis.

## Results

Indicator definitions and point estimates for 2012 and 2015 are shown in Table 2. The coverage of at least four antenatal care visits doubled (22% to 45%) and there was some evidence that antenatal care quality improved (measured by more women receiving recommended health worker behaviours for focussed antenatal care) as did coverage of two of the three lifesaving interventions iron supplementation (16% to 41%) and syphilis testing (8% to 13%). For delivery, the coverage of skilled attendance at birth more than doubled (18% to 51%) but the indicators of quality and intervention coverage presented a mixed picture. Improved birth attendant knowledge of management of heavy bleeding and increased availability of uterotonics were observed, both consistent with higher coverage of prophylactic uterotonics to prevent post-partum haemorrhage. Coverage of delayed bathing (37% to 54%) and immediate breastfeeding (44% to 62%) also increased. Conversely no change in birth attendant knowledge of immediate newborn care (thermal care) was observed and no increase in the coverage of immediate drying, wrapping, or clean cord care, although the percentage of newborns who had nothing harmful put on their cords improved. Coverage and quality of postnatal care for newborns within two days of birth remained very low and no change was observed for exclusive breastfeeding for the first three days of life, coverage of which was already high at baseline.

Results from the regression analysis of indicators of coverage of critical interventions on indicators of contacts and quality are shown in Tables 3, 4 and 5 and described below.

**Table 4:** Results from the multiple regression of change in coverage of critical interventions during pregnancy on change in indicators of contacts and quality.

	Change in coverage of tetanus toxoid protection		Change in coverage of iron supplementation		Change in coverage of syphilis detection	
	Coefficient (95% CI)	p value	Coefficient (95% CI)	p value	Coefficient (95% CI)	p value
Change in coverage of at least 4 ANC visits	0·17 (-0·12, 0·47)	0·24	0·00 (-0·29, 0·29)	0·99	-0·02 (-0·18, 0·14)	0·81
Change in focused ANC (behaviours delivered)	0·04 (-0·01, 0·09)	0·12	0·06 (0·01, 0·11)	0·02	0·07 (0·04, 0·10)	<0·001
Change in birth preparedness (number of items prepared)	0·02 (-0·11, 0·15)	0·78	0·09 (-0·04, 0·21)	0·18	0·02 (-0·05, 0·09)	0·52

**Table 5:** Results from the multiple regression of change in coverage of uterotonics on indicators of contacts and quality.

	Change in coverage of uterotonics	
	Coefficient (95% CI)	p value
Change in coverage of skilled attendant at birth	0·09 (-0·18, 0·36)	0·50
Change in knowledge about management of heavy bleeding	0·03 (-0·01, 0·07)	0·16
Change in availability of uterotonic supplies	0·72 (0·50, 0·94)	<0·001

### Pregnancy

In simple regression, for contacts, an increase in the proportion of women receiving four or more ANC contacts was associated with an increase in the coverage of all critical interventions in pregnancy; iron supplementation (regression coefficient (95% CI); 0·33 (0·10, 0·56)), tetanus toxoid prevention (0·37 (0·14, 0·60)), and syphilis testing (0·28 (0·13, 0·43)). This indicates that for every 10 percentage points increase in the coverage of women receiving four or more ANC contacts, the coverage of iron supplementation increased by 3·3 percentage points, the coverage of tetanus toxoid prevention by 3·7 percentage points and the coverage of syphilis testing by 2·8 percentage points.

In simple regression, for quality, there was evidence that both an increase in the number of health worker behaviours for focused ANC (Table 3) [14] and an increase in the number of items prepared for birth by the end of pregnancy were associated with an increase in the coverage of the critical interventions in pregnancy; iron supplementation (0·07 (0·04, 0·11); 0·19 (0·09, 0·29)), tetanus toxoid prevention (0·06 (0·02, 0·09); 0·12 (0·02, 0·23)) and syphilis testing (0·07 (0·05, 0·09); 0·14 (0·08, 0·21)).

In multiple regression of coverage of tetanus toxoid prevention (Table 4) on the change in contacts (the proportion of women receiving four or more ANC visits), the change in quality (number of health worker behaviours for focused ANC and the change in the number of items prepared for birth by the end of pregnancy) and baseline cluster level summaries, all associations were attenuated suggesting collinearity between the variables. The correlation between a change in the proportion of women who had at least four ANC visits and a change in the number of components of focused ANC received was 0·59; the correlation between a change in the proportion of women who had at least four ANC visits and a change in the number of items prepared for birth by the end of pregnancy was 0·26 and the correlation between a change in the two measures of quality was 0·45. The strongest remaining association was between an increase in contacts as measured by the proportion of women receiving four or more ANC visits (0·17 (-0·12, 0·47)) and change in coverage of tetanus toxoid protection.

In multiple regression of coverage of iron supplementation and syphilis prevention (Table 4) on the change in the proportion of women receiving four or more ANC visits, the change in the number of behaviours for focused ANC, the change in the number of items prepared for birth by the end of pregnancy and their baseline cluster level summaries, an increase in the quality indicator focused ANC behaviours remained associated with a change in both the coverage of iron supplementation and syphilis prevention (0·06 (0·01, 0·11); 0·07 (0·04, 0·10)).

### Intrapartum

In simple regression analysis for contacts, an increase in the number of deliveries with a skilled attendant at birth was associated with an increase in the coverage of delayed bathing (0·41 (0·22, 0·59)) and an increase in the coverage of uterotonics (0·61 (0·29, 0·93)), but not with an increase in the essential immediate newborn care indicators immediate breastfeeding, drying or wrapping (Table 3)

For quality, there was also evidence of an increase in knowledge about management of heavy bleeding amongst birth attendants and an increase in the availability of uterotonic supplies amongst birth attendants and both were associated with higher coverage of uterotonics (0·84 (0·66, 1·02); 0·09 (0·04, 0·14)) (Table 3).

In multiple regression of change in coverage of uterotonics on change in coverage of skilled attendant at birth, change in knowledge about management of heavy bleeding amongst birth attendants, change in the availability of uterotonic supplies for birth attendants and their baseline cluster level summaries, only the quality indicator change in the availability of uterotonic supplies amongst birth attendants remained associated with a change in the coverage of uterotonics (0·72 (0·50, 0·94)) (Table 5).

### Post-natal

There were no associations observed for either an increase in postnatal contacts or an increase in postnatal indicators of quality (a score composed from number of components of recommended post-natal care behaviours delivered (Table 2)) and an increase in coverage of postnatal critical interventions, however there was little scope for improvement in these indicators given the high coverage at baseline.

### Across stages along the continuum from pregnancy to postnatal

Similarly, no associations were observed between either an increase in ANC contacts or an increase in the number of recommended preparations a woman made for her delivery and either change in coverage of hand washing with soap by birth attendants (women delivering at home only), or change in the number of newborns with clean cord care.

## Discussion

This analysis aimed to determine the extent to which increased frequency or improved quality of contacts were associated with observed changes in intervention coverage for mothers and newborns in Ethiopia.

We found measures of quality to be consistently associated with coverage, but the relative contribution of increased number of contacts or improvements in quality to increases in coverage was not consistent across the different interventions examined.

In pregnancy, women who made more visits had higher coverage of tetanus toxoid protection independent of the quality indicators measured: this makes sense given that multiple ANC visits are needed to deliver multiple doses. But iron supplementation and syphilis testing, both of which can be provided at a single visit, had a stronger association with indicators of quality suggesting that women attending better quality facilities were more likely to receive these one-off interventions.

At delivery, a strong relationship emerged between more birth attendants having uterotonics available and improved coverage of prophylactic uterotonics to prevent post-partum haemorrhage, in the absence of any improvements in knowledge of their importance. In this example, successful delivery of the intervention is dependent on both availability of the commodity (input quality) and health worker use of it (process quality). The regression coefficient of 0·84 (0·72 adjusted) suggests that health worker behaviour was very good with respect to this aspect of care with a 0·8% increase in the proportion of women receiving a uterotonic for every 1% increase in the availability of uterotonics with the limiting factor being availability. Many lifesaving interventions at delivery rely on the behaviours of birth attendants rather than equipment or commodities, essential immediate newborn care providing good examples of this. Analysis of initiation of immediate breastfeeding, immediate drying and wrapping, and delayed bathing of the newborn revealed that only delayed bathing was associated with an increase in coverage of skilled attendance at birth, suggesting that, in 2015, the other behaviours had not yet become the accepted norm irrespective of health worker knowledge of them.

Finally, considerable missed opportunities were revealed for the delivery of lifesaving care across the continuum from pregnancy to newborn periods. We found no evidence of a pathway linking increased antenatal or delivery contacts to improved quality and intervention coverage within the early postnatal period, and coverage of early postnatal checks remained very low. In the context of increased facility delivery with early discharge, there is a need to re-evaluate the model for providing care to newborns within 2 days of birth to make sure they do not fall between community and facility structures.

This research was carried out across the four most populous regions of Ethiopia where the coverage of contacts, of quality, and the majority of interventions for mothers and newborns was suboptimal at the outset but showed evidence of marked improvement over the three year period. From a very low baseline, we observed improvement between 2012–2015 in the coverage and quality of antenatal and intrapartum care with changes of over 20 percentage points in some indicators such as the coverage of at least four antenatal care visits, the coverage of iron supplementation and the coverage of skilled attendants at birth. However immediate newborn interventions and the coverage and quality of postnatal care checks lagged behind Over a decade ago the government committed to increase demand for and availability of health services everywhere and, through a comprehensive mix of multi-sectoral strategies, made remarkable progress to this end [15]. A large number of external partners played a role in supporting the government, testing for example quality improvement initiatives [16], improving access to emergency transport [17] strengthening community linkages [18], and institutionalising maternal death surveillance and response systems [19]. Current government priorities, as described in the Health Sector Transformation Plan, clearly define targets to improve the quality, not only the quantity, of health care provided. This includes focussing on ensuring the basic foundations of health care, particularly important in the context of continued constraints in the availability of emergency obstetric care [20, 21], but also focussing on the quality of health worker behaviours. This commitment is highly consistent with the growing body of evidence that shows limited association between the availability of supplies for different health needs and appropriate usage by health workers [22]. The analysis presented here suggests that to achieve the national 2020 targets for maternal and newborn health the country will need both a catch up and keep up strategy–continuing to increase demand for contacts, continuing to strengthen and expand the foundations of the health system, while also improving the experience of health care users when they reach care.

A particular strength of the work was to evaluate the coverage of contacts and the quality of care provided simultaneously, as co-drivers for improving health, rather than consider these as independent constructs [23]. But a number of limitations exist. The analysis has change in coverage of lifesaving interventions as an endpoint, not mortality. While it is commonly understood that coverage is the essential penultimate step on the pathway towards improved survival [1, 12, 24] it provides less certainty than evidence of actual mortality impact. Analyses were conducted at the cluster level thus the ecological fallacy (potential mis-interpretation about the nature of individuals relative to the group to which those individuals belong) cannot be ruled out. Further, the analysis was predominantly driven by supply side factors (equipment and health worker behaviours) because many of the interventions examined were dependent on actions within health facilities: we did not account for the individual preferences of women and their families which may also have played a role in the intervention coverage. Measures of quality were illustrative not comprehensive. The World Health Organization has presented its vision of quality care for mothers and newborns [25] but there is little standardisation as yet around definitions and measures: the input and process quality hypotheses tested here are plausible but we acknowledge that they could have been constructed differently. Further, we used some proxy measures for quality, including linking frontline worker data to household observations to compensate for information about quality that mothers were not able to provide [26]. Finally, whilst we believe that the associations that remained significant in the multiple regression analysis are plausible and relevant to public health we carried out a large number of statistical tests and made no adjustment for multiple testing which may have led to false positive findings. We therefore urge cautious interpretation.

## Conclusion

This analysis of change in Ethiopia between 2012 and 2015 provides three important pieces of evidence for action. First, improvements in the quality of care were independently associated with increased coverage of critical antenatal and intrapartum interventions, supporting the current commitment to invest in quality [27, 28]. Second, that investment in quality needs to go beyond the inputs of the health service in terms of supplies and commodities and also include mechanisms for supporting the process of care through best practice by health workers. Third, that urgent attention is required to improve postnatal care, and more focus is needed on care that integrates opportunities for both the mother and newborn.

## Supporting information

S1 File Prospective analysis plan. (DOCX)
