# Supplementary material for: Associations between increased intervention coverage for mothers and newborns and the number and quality of contacts between families and health workers: An analysis of cluster level repeat cross sectional survey data in Ethiopia
Source: PLoS One. 2018 Aug 2;13(8):e0199937. doi: 10.1371/journal.pone.0199937 (PMC6071969; doi:10.1371/journal.pone.0199937)
Supplement: S1 File — (DOCX) [file pone.0199937.s001.docx]

**IDEAs objective 2 Statistical Analysis Plan – April 2015**

**Ethiopia**

**The analytical approach was simplified before carrying out the final analysis and the indicators were reviewed and revised. No of these changes were data driven.**

1. **Introduction**

This statistical analysis plan focusses on the Bill & Melinda Gates foundation maternal and newborn health theory of change. This theory of change hypothesises that where Innovations are put in place to enhance Interactions (make them more frequent, better quality, and equitable in reach), the coverage of life saving Interventions will increase, and more mothers and newborns will survive as a result. Within the IDEAS project the exploration of the relationship between Interactions and Interventions is referred to as learning question 2.


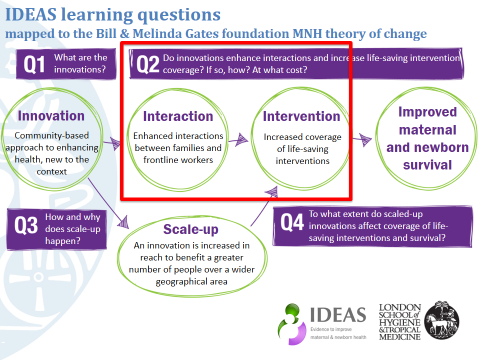


Learning question 2: To what degree do community-based maternal & newborn health innovations enhance interactions between families and frontline workers, and intervention coverage, in districts where implementation grantees work directly? Through what mechanisms do enhanced interactions (i.e. more, better and equitable) affect coverage of critical interventions?

Objective: To gather, analyse and synthesise evidence, in districts where grantees work directly, to determine whether community-based maternal and newborn health innovations lead to enhanced interactions between families and front line workers, and whether and how these lead in turn to increased intervention coverage.

The research is focused on areas of direct grantee activity, specifically where grantees are putting in place community based demand and supply side innovations for mothers and newborns (referred to by IDEAS as *key districts*).

The study design is a before-after “plausibility” comparison^[[1]](#footnote-1)^ of interactions between families and front line workers, and of intervention coverage, in both intervention (*key districts*) and comparison areas, between 2012 (baseline) and 2015 (follow-up).

The surveys are designed to measure:

1. the number of interactions between families and providers of maternal and newborn health services in communities and in health facilities;
2. the quality of those interactions, assessed on the basis of the equipment and commodities available to deliver maternal and newborn care at the time of survey, on the activities carried out by front line workers during the interactions, on the information delivered to and retained by service users, and on how timely the interactions are;
3. the coverage of critical interventions for mothers and newborns;
4. equity dimensions: the number and quality of interactions, and coverage of interventions, disaggregated by socio-economic status of households, ethnicity, and geographic position of households relative to health facilities.

Two levels of analysis will be carried out. First, indicators of the number and quality of interactions, and of intervention coverage, will be tabulated in intervention and comparison areas, and a difference-in-differences estimation of percentage point change between 2012 and 2015 carried out. Second, a set of hypotheses relating to the theory of change will be tested using regression analysis.

1. **Sampling and sample size**

**2.1 Sampling clusters**

Ethiopia is organised by region, zone, *woreda* (district), *kebele* (similar to a ward; lowest level of census population data) and *gote* (proxy for village). The baseline survey included 40 intervention (*key district)* and 40 comparison clusters, a cluster being defined as all households within a segmented *gote*.

Multi-stage cluster sampling was carried out. The 40 intervention clusters were sampled from 59 *woreda* across four regions of Ethiopia (Amhara, Oromia, Southern Nations Nationalities and People’s Region (SNNPR) and Tigray) where the project ‘Last 10 Kilometres’ (L10K) had already implemented an innovation to *anchor community health promoters in communities and to provide technical management support to community based organizations*, and where L10K planned an expansion of their programme of innovations throughout 2012-14. All 59 *woreda* were listed geographically from north to south of the country, and *kebeles* and their population size listed alphabetically within each *woreda;* 40 *kebele* were then sampled with probability proportional to population size. *Gotes* within each of these 40 *kebele* were subsequently listed and one *gote* per *kebele* selected using simple random sampling. At each selected *gote*, all households were listed and *gotes* segmented into groups of 75 or fewer households: field teams randomly selected one segment from each *gote* as the cluster to be surveyed.

The sampling frame for the 40 comparison clusters was defined by listing all *woreda* in the same zones as selected intervention clusters, excluding those *woreda* with any known BMGF funded activity in 2012, and repeating the multi-stage sampling approach applied for intervention clusters. In the final sample, there were five intervention and four comparison clusters in Tigray region, 15 intervention and 16 comparison clusters in Amhara region, 12 intervention and 16 comparison clusters in Oromia Region, and 8 intervention and 4 comparison clusters in SNPR.

**2.2 Sampling for the household interviews**

All households within each selected cluster were visited and a full household listing carried out. All resident women aged 13-49 were interviewed, with a detailed module applied for women with a birth in the 24 months prior to survey^[[2]](#footnote-2)^.

At the planning stage, the minimum target number of households per cluster was set at 50, meaning a minimum total number of 2000 households would be surveyed and an expected minimum number of 200 women with a live birth in the previous 12 months interviewed in both intervention and comparison areas^^[[3]](#footnote-3)^^. (In Ethiopia where the total fertility rate is estimated to be 4.8^^[[4]](#footnote-4)^^, we estimate that a cross-sectional survey would find one woman aged 13-49 who had a live birth in the previous 12 months in 10% of all households).

**2.3 Sampling for the frontline worker and facility interviews**

For each sampled cluster, the community level volunteers were identified and listed and a simple random sample of up to 3 volunteers selected for interview about recent health care they had provided. The health post assigned to the cluster was surveyed, and the health extension worker on duty interviewed. The primary health centre assigned to provide routine antenatal, intra-partum and post-natal care to the selected cluster was also surveyed, and the nurse who attended the last delivery recorded in the maternity register interviewed. Finally, the referral facility for each health centre was visited and a record review from maternity registers carried out to obtain information about caesarean sections.

**2.4 Power**

1. Power to detect percentages point change between 2012 and 2015 between intervention and comparison areas.

In planning the baseline survey, using an illustrative range of indicators along the continuum of care from antenatal to postpartum care and assuming a design effect of 1.4, we estimated that a sample of 2000 households and 200 women with a recent birth would have 90% power to measure changes of between 15 and 20 percentage points between baseline and endline across the entire intervention or comparison area.

Subsequent analysis of the baseline data suggests that, the baseline estimates were accurate but that the design effect is closer to 3 which would require the following numbers of births in the last 12 months. Sample sizes of under 2,000 families (households) are shown in ***bold.***

|  |  |  | **80% power** | | |
| --- | --- | --- | --- | --- | --- |
| **Area of investigation** | **Sample Indicator** | **Expected level at baseline** | **Sample size* (births in last 12 months) to detect percentage point increases of:** | | |
|  |  |  | **10** | **15** | **20** |
| **Frequency of interactions** | Attended ANC 4 or more times during pregnancy | 24% | 1086 | 504 | 294 |
|  | Delivery in a health facility | 10% | 660 | 332 | ***208*** |
|  | PNC within 48 hours | 6% | 504 | 268 | ***174*** |
| **Quality of interactions** | Pregnancy care from a skilled provider (inc HEW) | 64% | 1108 | 469 | ***249*** |
|  | Assistance at delivery from a trained health professional (inc HEW) | 14% | 797 | 388 | ***236*** |
| **Coverage of interventions** | TT coverage | 44% | 1292 | 574 | 321 |
|  | Babies breastfed within 1h of birth | 51% | 1275 | 559 | 309 |

1. Power to test hypotheses using regression analysis

Simulations have been carried out to explore the sample size requirements in terms of number of clusters (n=40) and effective sample size per cluster (n=5), assuming an increase of 3.5 (SD 0.3) in mean number of interactions from 1.5 at baseline to 5 interactions at endline, and the coefficients for the relationship between interactions and coverage of alpha (0.2) and beta (0.6).

**Simulation results**

Number of simulations = 1000

Number of clusters = 40

Number of women per cluster = 5

True coefficient for coverage-interaction relationship at baseline = .6

True coefficient for coverage-interaction relationship at endline = .6

True mean change in interactions = 3.5

Power

Percentage of studies P < 0.05 = **90.4 %**

1. **Data Sets**

Data will be collected at three levels: women within households, frontline workers serving those households, and primary health facility allocated to provide routine MNH to those households. The primary statistical analysis will be carried out at the cluster level.

1. **Demographic and Other Characteristics**

Baseline demographic and other characteristics will be tabulated. Descriptive statistics for continuous variables will include the mean, standard deviation, median, range and the number of observations. Categorical variables will be presented as numbers and percentages.

1. **Analysis**

Core indicators are defined to represent priority interactions between families and frontline workers (Table 1), measures of interaction quality (Table 2), and life saving interventions (Table 3). An expanded list of indicators is shown in the appendix. Summary data will be produced for these core (plus additional) indicators at both time points by area (comparison/intervention) and by cluster (Tables A1, A2 and A3). Analysis of rare outcomes will be restricted to descriptive analysis.

Table 1: Core indicators of priority interactions ‘more’

| **Pregnancy** |
| --- |
| Mean number of pregnancy care interactions reported by women |
| Percentage of women who had at least 4 pregnancy care interactions |
| **Intra-partum** |
| Percentage of women who were attended by a skilled attendant at birth |
| **Post-partum and post-natal** |
| Percentage of women who had a PPC within 2 days |
| Percentage of women who reported their newborn had a PNC within 2 days |

Table 2: Core indicators of interaction quality ‘better’

| **Pregnancy** |
| --- |
| Percentage of women who reported receiving good quality antenatal care by the end of pregnancy (received 7 components of focused antenatal care; made first PHC visit prior to 20 weeks gestation) |
| Percentage of women who made appropriate preparations for their delivery while still pregnant (5 items of recommended birth preparedness) |
| Percentage of women who had knowledge of danger signs during pregnancy |
| **Intra-partum** |
| Percentage of women who reported receiving good quality intrapartum care (delivered with a SBA who prepared all essential commodities; delivered in a facility where all essential commodities were present on day of survey) |
| Percentage of women who had knowledge of danger signs during delivery |
| **Post-partum and post-natal** |
| Percentage of women who reported receiving good quality PPC (received 5 components of recommended PPC) |
| Percentage of women who reported receiving good quality PNC (received 5 components of recommended PNC) |
| Percentage of women who had knowledge of danger signs for the newborn after delivery |

Table 3: Core indicators of critical life-saving interventions

| **Pregnancy** |
| --- |
| TT vaccination |
| Iron supplementation |
| Syphilis prevention and management (test results received) |
| **Intra-partum** |
| Prophylactic uterotonic immediately after birth  Use of gloves by birth attendant |
| **Newborn** |
| Clean cord care (cutting, tying, put nothing on) |
| Thermal care (immediate skin to skin, delayed bathing (>24hrs)) |
| Breastfeeding (immediate (<1hr)/exclusive 3 days) |

**5.1 Estimation of percentage point change between 2012 and 2015 in indicators of number and quality of interactions, and intervention coverage.**

To estimate the difference in change in percentage points of indicators between intervention and comparison areas from baseline to endline a difference in differences approach will be used. This will be estimated using linear regression at the cluster level with an interaction term between time and area (comparison/intervention). (Tables A1-A3)

We will estimate both the unadjusted effect of the area (comparison/intervention) on the indicators and an adjusted effect. Adjustment for contextual variables including other projects, urban/rural, region will be made in the primary analysis. Other information available on contextual factors will be use qualitatively when interpreting the results.

We will carry out sensitivity analyses excluding areas where other projects (eg CBNC) are active.

**5.2 Testing hypotheses around the BMGF theory of change.**

We will address the hypotheses displayed in Figures 1a-c below.

The impact of change between baseline and endline in the indicators of ‘more’ (Table 1) on both change between baseline and endline in the indicators of ‘better’ (Table 2) and change between baseline and endline in the ‘coverage of critical interventions’ (Table 3) will be assessed across all clusters using linear regression, regressing the cluster level mean difference in indicators of ‘more’ on the cluster level mean difference in ‘better’ or ‘coverage indicators’ between baseline and endline. The analysis will adjust for area (comparison/intervention). Results will be displayed graphically (Figure 2).

Figure 2 (illustrative data). Impact of cluster level change of number of antenatal care visits on change in coverage of TT vaccine, between baseline and endline

Figure 1a Pregnancy care

An increase in the coverage of critical post-natal (post-partum) interventions

An increase in the coverage of critical intra-partum interventions

Increases in the proportion of women who receive good quality intra-partum care

Increases in the number of intra-partum care interactions

An increase in the coverage of critical interventions in pregnancy

Increases in the proportion of women who receive good quality pregnancy care

Increases in the number of pregnancy care interactions

Figure 1b Intra-partum care

Figure 1c Post natal (infant) and post partum (mother) care

Increases in the number of post-natal (post-partum) care interactions

Increases in the proportion of women who receive good quality post-natal (post-partum) care

Further exploratory analysis will be undertaken to assess whether the impact of the change in indicators of ‘more’ differs by area (comparison/intervention) by fitting a model with an interaction between area (comparison/intervention) and indicators of ‘more’. Results will be displayed graphically (Figure 3).

Causal modelling will be used to explore the direction of any relationships.

Figure 3 (illustrative data). Impact of cluster level change of number of antenatal care visits on change in coverage of TT vaccine, between baseline and endline, in intervention and comparison areas separately

**5.3 Equity**

Principal components analysis will be used to derive an estimate of socio economic status. The change in the level of coverage of indicators will be tabulated by SES, baby’s gender (for newborn indicators) and mother’s literacy at the area (intervention/comparison) level.

Analyses at the individual level will be carried out to further explore the questions outlined in sections 5.1 and 5.2. Interaction terms between measures of equity and area (comparison/intervention) will be included in individual level models (linear and logistic) to assess the level to which the impact of equity status on the outcomes differs by area (comparison/intervention). Where the outcomes are continuous cluster level summaries of the baseline measure of the outcomes will be included in the models, however for binary outcomes, due to the non collapsibility of odds ratios we will not adjust for baseline summaries. All models will include a random effect for cluster.

**Appendix 1**

Table A1: Expanded indicators of MORE

|  | **Time** | **Comparison** | **Intervention** | **Unadjusted intervention effect (95% CI)** | **Adjusted intervention effect (95% CI)** |
| --- | --- | --- | --- | --- | --- |
| **Pregnancy** | | | | | |
| Mean number of pregnancy care interactions reported by women | Baseline |  |  |  |  |
|  | Follow up |  |  |  |  |
| Percentage of women who had an antenatal care visit with a skilled provider | Baseline |  |  |  |  |
|  | Follow up |  |  |  |  |
| Percentage of women who had at least 4 pregnancy care interactions | Baseline |  |  |  |  |
|  | Follow up |  |  |  |  |
| Percentage of women who had at least 1 home visit [disaggregated by FLW] | Baseline |  |  |  |  |
|  | Follow up |  |  |  |  |
| Percentage of women who attended a health facility at least once [disaggregated by level of facility] | Baseline |  |  |  |  |
|  | Follow up |  |  |  |  |
| Percentage of women who had at least 1 danger sign who sought care outside the home | Baseline |  |  |  |  |
|  | Follow up |  |  |  |  |
| **Intra-partum** | | | | | |
| Percentage of women who gave birth in a health facility | Baseline |  |  |  |  |
|  | Follow up |  |  |  |  |
| Percentage of women who were attended by a skilled attendant | Baseline |  |  |  |  |
|  | Follow up |  |  |  |  |
| Percentage of women who were referred to and attended higher care | Baseline |  |  |  |  |
|  | Follow up |  |  |  |  |
| **Post-partum** | | | | | |
| Percentage of women with a PPC within 2 days | Baseline |  |  |  |  |
|  | Follow up |  |  |  |  |
| Percentage of women with a PPC within 7 days | Baseline |  |  |  |  |
|  | Follow up |  |  |  |  |
| Percentage of women with a PPC within 7 days [disaggregated by FLW] | Baseline |  |  |  |  |
|  | Follow up |  |  |  |  |
| Percentage of women who had at least 1 danger sign who sought care outside the home | Baseline |  |  |  |  |
|  | Follow up |  |  |  |  |
| Percentage of women who reported their newborn had a PNC within 2 days | Baseline |  |  |  |  |
|  | Follow up |  |  |  |  |
| Percentage of women who reported their newborn had a PNC within 7 days | Baseline |  |  |  |  |
|  | Follow up |  |  |  |  |
| Percentage of women who reported their newborn had a PNC within 7 days [disaggregated by FLW] | Baseline |  |  |  |  |
|  | Follow up |  |  |  |  |
| Percentage of women who reported their newborn had at least 1 danger sign who sought care outside the home | Baseline |  |  |  |  |
|  | Follow up |  |  |  |  |

Table A2: Expanded list of indicators of BETTER

|  | **Time** | **Comparison** | **Intervention** | **Unadjusted intervention effect (95% CI)** | **Adjusted intervention effect (95% CI)** |
| --- | --- | --- | --- | --- | --- |
| **Pregnancy** | | | | | |
| Mean (median) gestation weeks at first pregnancy care interaction reported | Baseline |  |  |  |  |
|  | Follow up |  |  |  |  |
| Percentage of women receiving good quality antenatal care^[[5]](#footnote-5)^ | Baseline |  |  |  |  |
|  | Follow up |  |  |  |  |
| Percentage of women with knowledge of pregnancy danger signs | Baseline |  |  |  |  |
|  | Follow up |  |  |  |  |
| Percentage of women who prepared for birth^[[6]](#footnote-6)^ while still pregnant | Baseline |  |  |  |  |
|  | Follow up |  |  |  |  |
| Percentage of facilities with essential ANC commodities^[[7]](#footnote-7)^ | Baseline |  |  |  |  |
|  | Follow up |  |  |  |  |
| Percentage of FLWs who with knowledge of pregnancy care^[[8]](#footnote-8)^ | Baseline |  |  |  |  |
|  | Follow up |  |  |  |  |
| **Intra-partum** | | | | | |
| Percentage of women receiving good quality intrapartum care* | Baseline |  |  |  |  |
|  | Follow up |  |  |  |  |
| Percentage of women who had knowledge of delivery danger signs | Baseline |  |  |  |  |
|  | Follow up |  |  |  |  |
| Percentage of facilities with commodities* for intrapartum care | Baseline |  |  |  |  |
|  | Follow up |  |  |  |  |
| Percentage of FLWs who had appropriate knowledge of intra-partum care* | Baseline |  |  |  |  |
|  | Follow up |  |  |  |  |
| **Post-partum** | | | | | |
| Percentage of women who reported receiving good quality PPC* | Baseline |  |  |  |  |
|  | Follow up |  |  |  |  |
| Percentage of FLWs who had knowledge of appropriate PPC* | Baseline |  |  |  |  |
|  | Follow up |  |  |  |  |
| Percentage of women who reported receiving good quality PNC* | Baseline |  |  |  |  |
|  | Follow up |  |  |  |  |
| Percentage of women who had knowledge of danger signs for the newborn after delivery | Baseline |  |  |  |  |
|  | Follow up |  |  |  |  |
| Percentage of facilities that had the essential commodities* available to deliver PNC | Baseline |  |  |  |  |
|  | Follow up |  |  |  |  |
| Percentage of FLWs who had knowledge of appropriate PNC* | Baseline |  |  |  |  |
|  | Follow up |  |  |  |  |

Table A3: Expanded list of critical life saving interventions

|  | **Time** | **Comparison**  **n(%)** | **Intervention**  **n(%)** | **Unadjusted intervention effect (95% CI)** | **Adjusted intervention effect (95% CI)** |
| --- | --- | --- | --- | --- | --- |
| **Pregnancy** | | | | | |
| TT vaccination | Baseline |  |  |  |  |
|  | Follow up |  |  |  |  |
| Iron supplementation | Baseline |  |  |  |  |
|  | Follow up |  |  |  |  |
| IPTp (2 doses) | Baseline |  |  |  |  |
|  | Follow up |  |  |  |  |
| Personal use of ITNs | Baseline |  |  |  |  |
|  | Follow up |  |  |  |  |
| Syphilis prevention and management | Baseline |  |  |  |  |
|  | Follow up |  |  |  |  |
| **Intra-partum** | | | | | |
| Prophylactic uterotonics to prevent PPH | Baseline |  |  |  |  |
|  | Follow up |  |  |  |  |
| Active management of third stage labour | Baseline |  |  |  |  |
|  | Follow up |  |  |  |  |
| Hand washing with soap by delivery attendant | Baseline |  |  |  |  |
|  | Follow up |  |  |  |  |
| Caesarean sections | Baseline |  |  |  |  |
|  | Follow up |  |  |  |  |
| Use of gloves by delivery attendant | Baseline |  |  |  |  |
|  | Follow up |  |  |  |  |
| **Newborn** | | | | | |
| Clean cord care (cutting, tying, put nothing on) | Baseline |  |  |  |  |
|  | Follow up |  |  |  |  |
| Thermal care (drying, wrapping, skin to skin, delayed bathing) | Baseline |  |  |  |  |
|  | Follow up |  |  |  |  |
| Detection and appropriate management of complications (infection, respiratory, LBW, prematurity) | Baseline |  |  |  |  |
|  | Follow up |  |  |  |  |
| Breastfeeding (immediate/exclusive 3 days) | Baseline |  |  |  |  |
|  | Follow up |  |  |  |  |
| **Post-partum** | | | | | |
| Detection and treatment of anaemia | Baseline |  |  |  |  |
|  | Follow up |  |  |  |  |
| Detection and treatment of post partum sepsis | Baseline |  |  |  |  |
|  | Follow up |  |  |  |  |

1. see Evaluation designs for adequacy, plausibility and probability of public heath performance and impact. JP Habicht, CG Victora and JP Vaughan. International Journal of Epidemiology 1999; 28: 10-18 [↑](#footnote-ref-1)
2. Data collected for births in last 24 months, but analysis described here restricted to births in previous 12 months [↑](#footnote-ref-2)
3. A separate document “Ethiopia baseline survey manual” describes the survey methodology in full [↑](#footnote-ref-3)
4. Ethiopia DHS preliminary report 2011 [↑](#footnote-ref-4)
5. Blood pressure checked; Urine and blood tested; counselled on breast feeding, danger signs, birth preparedness; weight and height measured. [↑](#footnote-ref-5)
6. Prepared finances, transport, identified a birth attendant, and a facility, and a blood donor, prepared materials for clean delivery (clean birth kit: clean cloth, cover to deliver on, gloves, cotton gauze) [↑](#footnote-ref-6)
7. Examination bed, stethoscope, sphygmomanometer, thermometer, weighing scale (adult), measuring tape, fetostethoscope, watch/timing device, disposable gloves, height stick, test kits. Plus TT vaccine, single use syringes/needles, ferrous + folic acid, sufadoxine pyrimethamine (in malaria areas) [↑](#footnote-ref-7)
8. minimum of 4 consultations, ensure there is a birth plan, prevention of illness (TT vaccine, iron, malaria), identify and manage infections (e.g. RTI/STI), teach danger signs, promote breastfeeding [↑](#footnote-ref-8)
